# De novo sequencing, assembly and functional annotation of *Armillaria borealis* genome

**DOI:** 10.1186/s12864-020-06964-6

**Published:** 2020-09-10

**Authors:** Vasilina S. Akulova, Vadim V. Sharov, Anastasiya I. Aksyonova, Yuliya A. Putintseva, Natalya V. Oreshkova, Sergey I. Feranchuk, Dmitry A. Kuzmin, Igor N. Pavlov, Yulia A. Litovka, Konstantin V. Krutovsky

**Affiliations:** 1 Laboratory of Forest Genomics, Genome Research and Education Center, Institute of Fundamental Biology and Biotechnology, Siberian Federal University, 660036 Krasnoyarsk, Russia; 2 Laboratory of Genomic Research and Biotechnology, Federal Research Center “Krasnoyarsk Science Center of the Siberian Branch of the Russian Academy of Sciences”, 660036 Krasnoyarsk, Russia; 3 Department of High Performance Computing, Institute of Space and Information Technologies, Siberian Federal University, 660074 Krasnoyarsk, Russia; 4 Laboratory of Forest Genetics and Selection, V. N. Sukachev Institute of Forest, Siberian Branch of Russian Academy of Sciences, 660036 Krasnoyarsk, Russia; 5 Department of Informatics, National Research Technical University, 664074 Irkutsk, Russia; 6 Limnological Institute, Siberian Branch of Russian Academy of Sciences, 664033 Irkutsk, Russia; 7 Laboratory of Reforestation, Mycology and Plant Pathology, V. N. Sukachev Institute of Forest, Siberian Branch of Russian Academy of Sciences, 660036 Krasnoyarsk, Russia; 8 Department of Chemical Technology of Wood and Biotechnology, Reshetnev Siberian State University of Science and Technology, Krasnoyarsk, 660049 Russia; 9 Department of Forest Genetics and Forest Tree Breeding, Georg-August University of Göttingen, 37077 Göttingen, Germany; 10 Center for Integrated Breeding Research, George-August University of Göttingen, 37075 Göttingen, Germany; 11 Laboratory of Population Genetics, N. I. Vavilov Institute of General Genetics, Russian Academy of Sciences, 119333 Moscow, Russia; 12 Department of Ecosystem Science and Management, Texas A&M University, College Station, TX 77843-2138 USA

**Keywords:** Annotation, *Armillaria*, De novo sequencing, Fungal forest pathogen, Genome

## Abstract

**Background:**

Massive forest decline has been observed almost everywhere as a result of negative anthropogenic and climatic effects, which can interact with pests, fungi and other phytopathogens and aggravate their effects. Climatic changes can weaken trees and make fungi, such as *Armillaria* more destructive. *Armillaria borealis* (Marxm. & Korhonen) is a fungus from the Physalacriaceae family (*Basidiomycota*) widely distributed in Eurasia, including Siberia and the Far East. Species from this genus cause the root white rot disease that weakens and often kills woody plants. However, little is known about ecological behavior and genetics of *A. borealis*. According to field research data, *A. borealis* is less pathogenic than *A. ostoyae*, and its aggressive behavior is quite rare. Mainly *A. borealis* behaves as a secondary pathogen killing trees already weakened by other factors. However, changing environment might cause unpredictable effects in fungus behavior.

**Results:**

The de novo genome assembly and annotation were performed for the *A. borealis* species for the first time and presented in this study. The *A. borealis* genome assembly contained ~ 68 Mbp and was comparable with ~ 60 and ~ 79.5 Mbp for the *A. ostoyae* and *A. mellea* genomes, respectively. The N50 for contigs equaled 50,544 bp. Functional annotation analysis revealed 21,969 protein coding genes and provided data for further comparative analysis. Repetitive sequences were also identified. The main focus for further study and comparative analysis will be on the enzymes and regulatory factors associated with pathogenicity.

**Conclusions:**

Pathogenic fungi such as *Armillaria* are currently one of the main problems in forest conservation. A comprehensive study of these species and their pathogenicity is of great importance and needs good genomic resources. The assembled genome of *A. borealis* presented in this study is of sufficiently good quality for further detailed comparative study on the composition of enzymes in other *Armillaria* species. There is also a fundamental problem with the identification and classification of species of the *Armillaria* genus, where the study of repetitive sequences in the genomes of basidiomycetes and their comparative analysis will help us identify more accurately taxonomy of these species and reveal their evolutionary relationships.

## Background

Massive forest decline as a result of negative anthropogenic and climatic effects, often aggravated by pests, fungi, and other phytopathogens, has been observed almost everywhere. Environmental changes, such as increased average annual temperatures, decreased precipitation, more frequent droughts, can weaken trees and make fungi much more destructive. Forest conservation has become a serious issue since the scale of plant death caused by phytopathogenic fungi is enormous. For instance, tree diseases have caused the loss of approximately 100 million elm trees in the United Kingdom and the United States, and the list can be continued. Among all phytopathogens, fungi cause 64% of infection-related species extinction and regional extirpation events [1].

The basidiomycete genus *Armillaria* plays a very important role in forest ecosystems worldwide and currently includes more than 40 officially described species [2, 3]. *Armillaria* species differ significantly in virulence, for example, some species, such as *A. ostoyae*, are the main cause of tree death while other species colonize plants already damaged by various factors (drought, pests, etc.) [4, 5]. Difference in pathogenicity has also been observed in *A. ostoyae*, however virulence variation of *A. borealis* has not been studied yet [6].

*Armillaria borealis* (Marxm. & Korhonen) is a fungus from the *Physalacriaceae* family (*Basidiomycota*) widely distributed in Eurasia, including Siberia and the Far East [1]. Species from this genus cause the root white rot disease that weakens and often kills woody plants [7]. Several phylogenetic and genomic studies on *A. ostoyae* have been carried out due to its high pathogenic potential and common occurrence [4, 8, 9], while little is known about ecological behavior of *A. borealis*. According to field research data, *A. borealis* is less pathogenic than *A. ostoyae*, and its aggressive behavior is rare. Mainly *A. borealis* behaves as a secondary pathogen killing trees already weakened by biotic and abiotic factors [10–13]. However, changing environment might cause unpredictable effects in fungi behavior.

*Armillaria spp.* impact on forest populations has both economic and ecological significance. They attack hundreds of different tree species (e.g., *Abies, Picea, Pinus, Betula, Sorbus, Juglans, Malus*, etc.) in both hemispheres under different climatic conditions, and are among the most destructive forest pathogens [2, 14, 15].

Identification of species and pathogenicity levels of *Armillaria* is crucial for forest conservation. The genomic data are needed to study the pathogenicity of pathogenic species and to better understand their impact on trees and the host-pathogen interactions. In addition, comparative genomics can help to resolve complex phylogeny of *Armillaria* species. It is worth noting that fungi genomic data are also important for industrial applications. For example, white rot *Armillaria* fungi are capable of lignin and cellulose decomposition, and they can be used to utilize the wood and paper production waste [16].

*A. borealis* is very important for the vast boreal forest ecosystems. However, despite the enormous influence of *Armillaria* species on forestry, horticulture, and agriculture, fungi of this genus and their pathogenicity are still not well-studied in this large region, which makes the presented genomic study very much needed.

There are already published genomic and proteomic data for *A. mellea*, *A. solidipes*, and *A. ostoyae* revealing the presence of plant cell wall degradation enzymes (PCWDE) and some secreted proteins [17–19]. Genomic analysis of other pathogenic basidiomycetes, such as *Moniliophthora* [20, 21], *Heterobasidion* [22], and *Rhizoctonia* [23], also revealed genes encoding PCWDE, as well as secreted enzymes and secondary metabolism effector proteins as putative pathogenicity factors. However, the life cycle and the distribution strategy of *Armillaria* members indicate that they may have evolved other additional mechanisms for pathogenicity, which along with other potential genomic mechanisms are not yet studied [24]. It is worth noting that the role and functional significance of mobile and highly repetitive elements (REs) are still not completely clear. Gradually accumulated data suggest that REs can play an important role in the evolutionary development of organisms, replication, and formation of nucleoprotein complexes, as well as affect gene expression [17]. Genomes of fungi are densely packed containing effector genes and transposable elements (TEs) [25–27]. It was reported that different fungal pathogens, such as *Fusarium* [28] and *Verticillium* [29], have similar genome architecture. So, it is expected that TEs may play important roles in host switching and adaptation to new ecological niches [30]. It was found in *Magnaporthe oryzae* that genes involved in host specialization were associated with TEs [31].

## Results

### Genome assembly

The *A. borealis* genome assembly contained ~ 67 Mbp and was comparable with ~ 60 and ~ 79.5 Mbp for the *A. ostoyae* and *A. mellea* genomes, respectively. The N50 for contigs equaled 50,554 bp (Table 1).

**Table 1:** Assembly parameters of the *Armillaria borealis* genome

Parameter	Contig
Number	44,412
Total length, bp	66,792,428
Maximum length, bp	2,136,877
N50, bp	50,544
N90, bp	346

### Completeness of the genome assembly

The BUSCO v3.1.0 software [32] was used to evaluate the completeness of the genome assembly. It showed that 94.7% of reference genes were captured as complete single-copy genes (Table 2). In addition, the RNA sequence reads were mapped to the genome assembly by TopHat v. 2.1.0 [33].

**Table 2:** Results of the *Armillaria borealis* genome assembly assessment using BUSCO

Genes	Number	Percentage
Complete (single-copy)	1286	96.3 (94.7)
Fragmented	17	1.3
Missing	32	2.4
Total number	1335	100

### Content of the RNA-seq reads

The SortMeRNA v2.1 program [34] with the pre-installed eight rRNA databases (silva-bac-16 s-id90, silva-arc-16 s-id95, silva-euk-18 s-id95, silva-bac-23 s-id98, silva-arc-23 s-id98, silva-euk-28 s-id98, rfam-5 s-id98, and rfam-5.8 s-id98) from the SILVA rRNA database project (SILVA SSU and LSU Ref NR v.119; https://www.arb-silva.de) was used to check the content of the RNA sequences. The results are presented in Table 3. Both RNA samples contained high number of rRNA reads – 32.5 and 72.9%, respectively.

**Table 3:** Content of the RNA-seq reads

Parameter	Sample 1	Sample 2
Total number of reads (%)	6,428,260 (100%)	8,444,300 (100%)
Number of non-rRNA reads (%)	4,339,634 (67.5%)	2,288,932 (27.1%)
Number of rRNA reads (%)	2,088,626 (32.5%)	6,155,368 (72.9%)
Average read length, bp	153	151

### Genome gene annotation

**Fig. 1:**
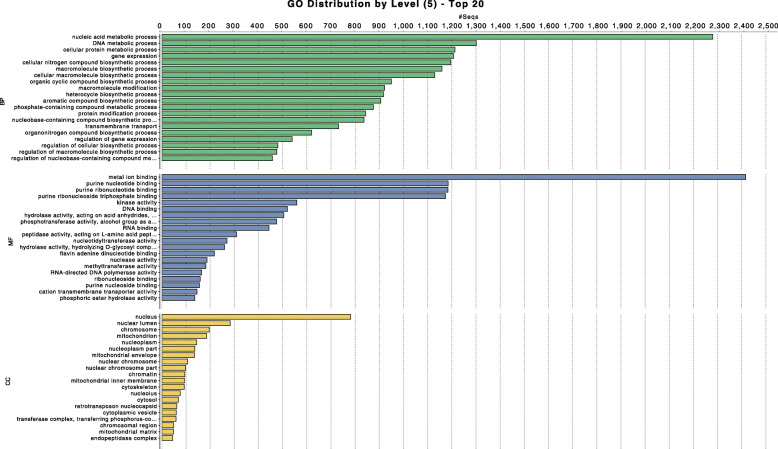
Distribution of 21,969 protein coding genes found in the *Armillaria borealis* genome assembly at the three levels - molecular function (MF), biological processes (BP), and cellular components (CC), respectively, based on the GO functional annotation

Functional annotation revealed 21,969 protein coding genes, which was also comparable with 22,705 and 14,473 genes in *A. ostoyae* and *A. mellea*, respectively. Their gene ontology (GO) functional annotation is presented in Fig. 1 and Additional files 1, 2 and 3. The greatest number of annotated sequences was related to the functioning of the cell nucleus (Fig. 1).

**Fig. 2:**
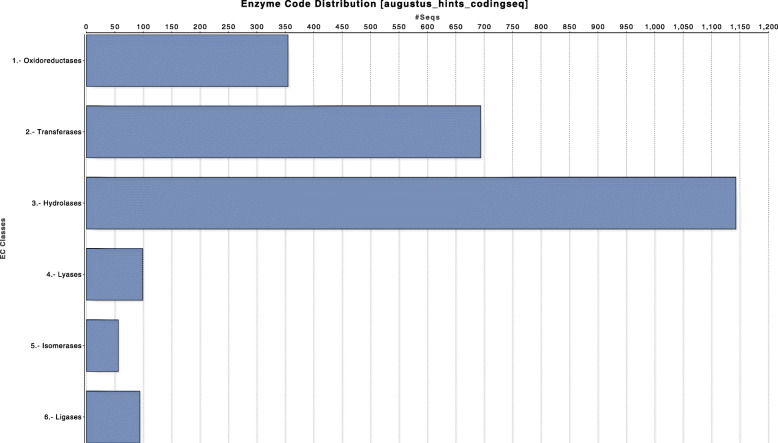
Distribution of enzyme genes found in the *Armillaria borealis* genome assembly across main classes

The distribution of enzyme genes across main classes is represented in Fig. 2. Oxidoreductases and hydrolases were among the most abundant enzymes.

### Repetitive element (RE) annotation

In total, 886 RE sequences were identified in the *A. borealis* genome assembly. However, 839 (94.7%) of them remained unrecognized based on the initial classification using RepeatModeler program (Table 4).

**Table 4:** Initial classification of the repetitive elements (REs) identified in the *A. borealis* genome assembly

RE type	RE family	Number
LTR retrotransposons	Copia	5
	Gypsy	32
	Pao	1
LINE-retrotransposons	Tad1	5
Retrotransposons with a tyrosine recombinase (YR)	Ngaro	3
Simple sequence repeats	Simple Repeats	1
**Classified**		**47 (5.3%)**
**Unclassified**		**839 (94.7%)**
**Total**		**886**

The following RE types were identified: LTR retrotransposons (Copia, Gypsy, Pao), LINE-retrotransposons (Tad1), and retrotransposons with a tyrosine recombinase (Ngaro).

The TEclass classifier software allowed us to further partition sequences including initially unclassified into four main groups (Table 5). The additional comparative studies of repetitive sequences in the genomes of basidiomycetes are needed to further classify REs. The variety of the detected TE families was not very wide, excep the LTR TE family, which was widely represented in *A. borealis*. The Ty3/Gypsy and Ty1/Copia elements have been identified, and Gypsy was the most abundant among them.

**Table 5:** Additional classification based on TEclass including initially unclassified repetitive elements (REs) identified in the *A. borealis* genome assembly

RE type	Number	%
DNA-transposons	79	9
Retrotransposons:		
LTR	735	83
LINE	27	3
Unclear	18	2
Total	886	100

## Discussion

Typical fungal nuclear genome sizes occupy an intermediate position between prokaryotes and other eukaryotes. On average, the size of the fungal genome is two orders of magnitude smaller than that of higher plants and varies from ~ 2.19 to ~ 3706 Mbp ( [35], see also DOE JGI Fungi Portal: https://mycocosm.jgi.doe.gov/mycocosm/home). The average genome size of *Ascomycota* and *Basidiomycota* divisions is ~ 36.91 and ~ 46.48 Mb, respectively [36]. The genome size of *A. borealis* (~ 66.79 Mbp) was within a range of genome sizes in closely related species. For example, the genomes of *A. ostoyae* and *A. mellea* were ~ 60 and ~ 79.5 Mbp, respectively [19, 37].

The genome sizes of our assembly and draft assembly of *Armillaria borealis* FPL87.14 v1.0 available in the US DoE JGI fungal genomics resource database (https://mycocosm.jgi.doe.gov/Armbor1) are quite comparable, ~ 66.8 vs. ~ 71.69 Mbp, respectively. The difference can be explained by the use of different sequencing technologies (Illumina MiSeq vs. PacBio) that resulted in significant differences in the number of scaffolds between two assemblies but did not influence much the number of coding sequences identified, 21,969 coding sequences in our assembly vs. 19,984 in *Armillaria borealis* FPL87.14 v1.0. Meanwhile, the genome size of *A. gallica* [19] is almost 19 Mbp bigger than in *A. borealis*. The number of genes identified in the *A. gallica* genome was also larger (~ 25,000 genes).

Enzymatic activities of plant cell wall degrading fungi are performed by complex mixtures of cellulases, hemicellulases, and ligninases [38]. Oxidoreductases and hydrolases are the most interesting among enzymes in *Armillaria* because they are involved in lignin (oxidoreductases) and cellulose (hydrolases) decomposition.

Among identified TE classes LTR elements were the most frequent, particularly in basidiomycete fungi [39]. It is also true for *A. borealis*. The effect of TE on lifestyle of white-rot fungi has not been studied in depth yet, but some studies revealed considerable difference in TE number among different *Armillaria* species [19]. Their detailed comparative analysis will be presented in a separate paper.

## Conclusions

The destruction of forests by pathogenic fungi is one of the main problems of forest conservation. Further comprehensive studies of these fungi at genomic, transcriptomic, proteomic, and metabolomic levels are very much needed to identify causes and mechanisms of their increasing pathogenicity. Our study provides important genomic resources of sufficiently good quality for further detailed work on studying the genetics of pathogenicity of *Armillaria* and other fungi species. It should also help with in-depth evolutionary and phylogenomic analyses and better identification and classification of *Armillaria* species genus.

## Methods

### Sample collection and DNA sequencing

The active mycelia of *A. borealis* were collected from dead trees of *Abies sibirica* in 2015 in a mixed forest consisted mainly of Siberian fir, silver birch, Norway spruce, and Siberian stone pine and located 40 km to the northwest of Krasnoyarsk City, Russia (56.175847°N, 92.184933°E). The fresh mycelium was isolated from under the bark of the infested stems 50 cm above the soil surface using sterile tweezers and gloves to avoid contamination. Before DNA extraction, mycelium was fixed for 2 days at 4°С in RNAlater (Thermo Fisher Scientific Company, Waltham, Massachusetts, USA). Then RNAlater fixed mycelium was quickly ground in acid-washed and autoclaved mortar. DNA was isolated using a modified version of the hot-CTAB extraction at 65 °C [40], followed by chloroform (double washing). Total DNA was precipitated for an hour with isopropanol at 4 °C, centrifuged at 6500 g for 30 min at 4 °C, washed twice with 70% ethanol, and was eluted in 50 μl nuclease-free water. Integrity and amount of the isolated total DNA were examined by 1.5% (wt/vol) agarose gel electrophoresis, and using the NanoDrop 1000 Spectrophotometer (Thermo Fisher Scientific Company, Waltham, Massachusetts, USA). The amount of extracted DNA was also measured on the Invitrogen Qubit 4 Fluorometer (Thermo Fisher Scientific Company, Waltham, Massachusetts, USA).

The paired-end sequencing libraries with 500 bp long genomic DNA inserts were generated using Truseq DNA Sample Prep Kit according to the manufacturer’s instructions (Illumina, Inc., San Diego, CA, USA). MiSeq Reagent Kit v2 (500-cycles) was used to sequence on the Illumina MiSeq platform with 2 × 250 cycles at the Laboratory of Forest Genomics of Siberian Federal University (Genome Research and Education Center, Siberian Federal University, Krasnoyarsk, Russia).

### RNA isolation and sequencing

Two samples were isolated from the active *A. borealis* mycelium from two dead trees of *Abies sibirica*, respectively, in 2015 and fixed for 2 days at 4°С in RNAlater (Thermo Fisher Scientific Company, Waltham, Massachusetts, USA). The distance between the trees was 2–10 m. The RNA was isolated using Qiagen RNeasy Mini Kit (Qiagen, Valencia, CA, USA).

The quality and concentration of the RNA were measured on Agilent 2100 Bioanalyzer using Agilent RNA 6000 Nano kit (Agilent Technologies, Inc., Santa Clara, CA, USA). Purified RNA with high quality was selected for further cDNA library construction. Purification of mRNA from total RNA was performed using Oligo (dT) magnetic beads. The mRNA treated with fragmentation buffer was used as a template for cDNA synthesis. A double-stranded cDNA library was constructed with the TruSeq RNA Library Prep Kit v2 (Illumina, Inc., San Diego, CA, USA). End-repair, A-tailing, adapter ligation, and library amplification were performed during cDNA library construction followed by cluster generation and sequencing on the Illumina MiSeq platform in the Laboratory of Forest Genomics (Genome Research and Education Center, Siberian Federal University, Krasnoyarsk, Russia) using MiSeq Reagent Kit v2 (2 × 150 cycles).

### Genome assembly

The de novo genome assembly was performed using SPAdes 3.13.0 genome assembler (http://cab.spbu.ru/software/spades) on high-performance computing (HPC) system IBM × 3950 X6 with 96 CPU and 3 TB RAM using an iterative genome assembly module for short reads. *K*-mer values were automatically selected based on read length and data type [41].

### Gene annotation

The gene annotation of the *A. borealis* genome was performed using BRAKER2 [42], which is a combination of GeneMark-ET [43] and AUGUSTUS [44], that uses genomic and RNA-Seq data to automatically generate full gene structure annotations in novel genomes. AUGUSTUS integrates the extrinsic evidence from protein homology information into the prediction. There were no protein data for *A. borealis* before this study, therefore protein sequences of a close relative *A. ostoyae* have been used [19].

The gene ontology (GO) functional annotation was carried out using Blast2GO [45]. This program worked directly with coding sequences in fasta format. First, the nucleotide sequences homologues to the *A. borealis* sequences were searched for in the BLAST database. Then, sequence mapping and annotation were carried out. In parallel, protein domains were detected using InterProScan [46].

### Repetitive element (RE) annotation

Repetitive sequences were identified initially using the RepeatModeler program designed to perform de novo search [47]. The additional classification of RE sequences was done using TEclass program [48].

## Supplementary information

**Additional file 1: Figure S1.** GO distribution of coding sequences found in the *Armillaria borealis* genome assembly at the biological processes (BP) level based on the GO functional annotation.

**Additional file 2: Figure S2.** GO distribution of coding sequences found in the *Armillaria borealis* genome assembly at the molecular function (MF) level based on the GO functional annotation.

**Additional file 3: Figure S3.** GO distribution of coding sequences found in the *Armillaria borealis* genome assembly at the cellular components (CC) level based on the GO functional annotation.

## Data Availability

The *A. borealis* genome assembly and all sequences described in this study are available in GenBank under the accession number JAAGUC000000000, sample SAMN13920850, project PRJNA603128 (https://www.ncbi.nlm.nih.gov/bioproject/603128).

## Abbreviations

BLAST: Basic Local Alignment Search Tool
bp: Base pair
CPU: Central processing unit
DNA: Deoxyribonucleic acid
LINE: Long interspersed nuclear element
LTR: Long terminal repeat
Mbp: Million base pair
N50: A weighted median statistic such that 50% of the entire assembly is contained in contigs or scaffolds equal to or larger than this value
N90: A weighted median statistic such that 90% of the entire assembly is contained in contigs or scaffolds equal to or larger than this value
PCWDE: Plant cell wall degradation enzymes
RAM: Random access memory
RE: Repetitive element
RNA: Ribonucleic acid
rRNA: ribosomal RNA
SINE: Short interspersed nuclear element
TE: Transposable element
