# Supplementary figures and images for: De novo sequencing, assembly and functional annotation of Armillaria borealis genome
Source: BMC Genomics. 2020 Sep 10;21(Suppl 7):534. doi: 10.1186/s12864-020-06964-6 (PMC7487993; doi:10.1186/s12864-020-06964-6)

Direct GO Count (BP) [augustus\_hints\_codingseq]

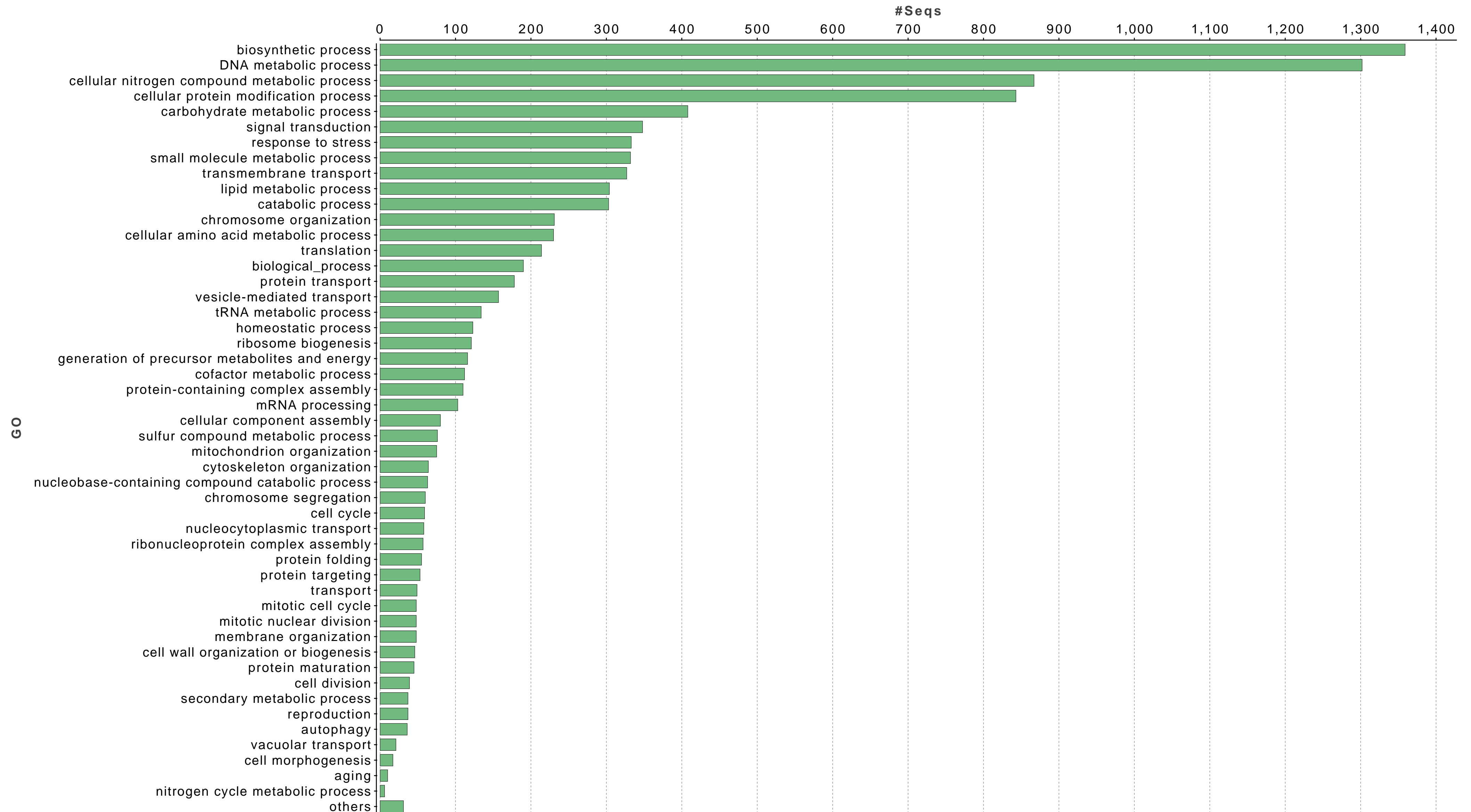

Supplement: Supplementary file 1 — Additional file 1: Figure S1. GO distribution of coding sequences found in the Armillaria borealis genome assembly at the biological processes (BP) level based on the GO functional annotation. [file 12864_2020_6964_MOESM1_ESM.pdf]

Direct GO Count (CC) [augustus\_hints\_codingseq]

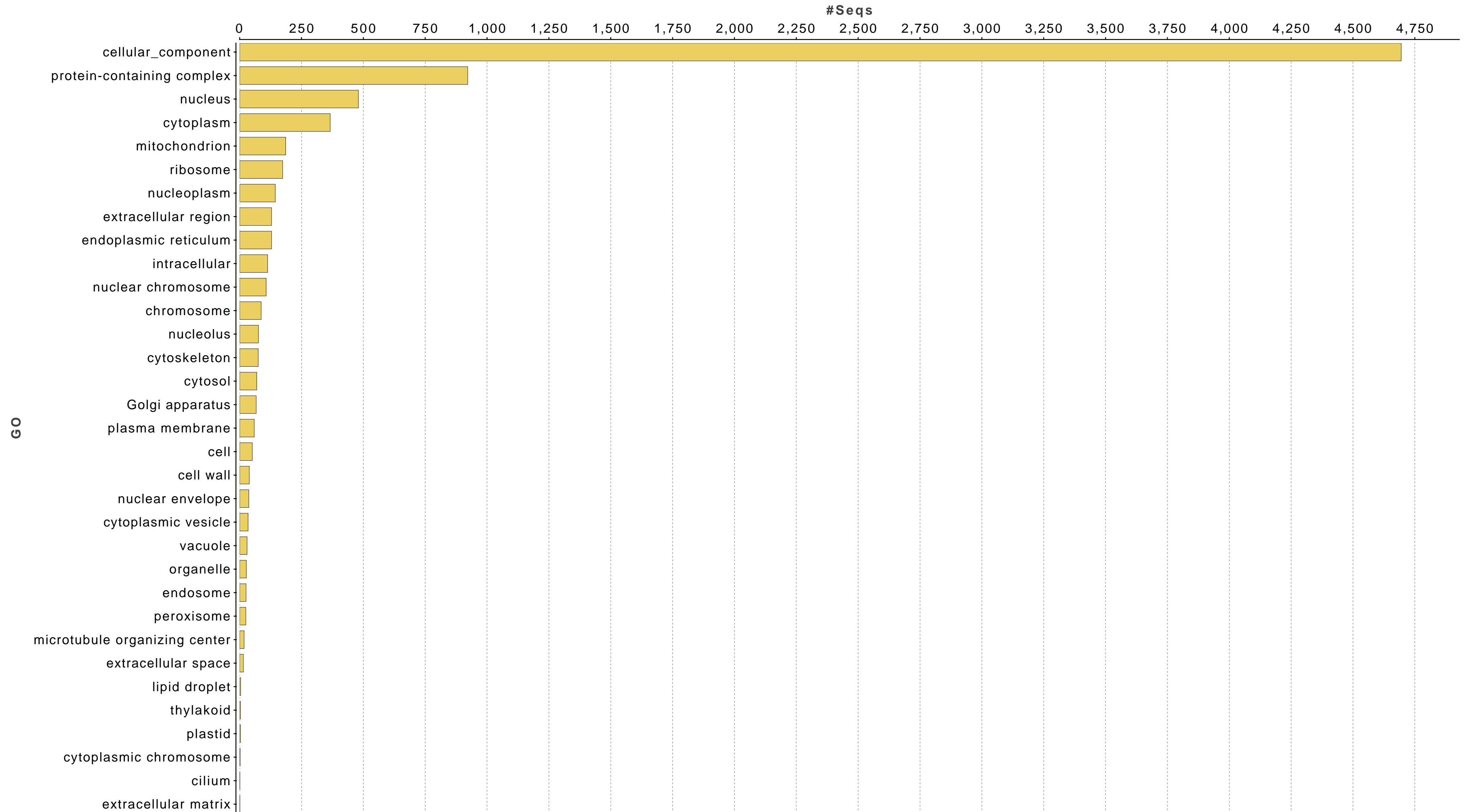

Supplement: Supplementary file 3 — Additional file 3: Figure S3. GO distribution of coding sequences found in the Armillaria borealis genome assembly at the cellular components (CC) level based on the GO functional annotation. [file 12864_2020_6964_MOESM3_ESM.pdf]
