# Supplementary material for: De novo sequencing, assembly and functional annotation of Armillaria borealis genome
Source: BMC Genomics. 2020 Sep 10;21(Suppl 7):534. doi: 10.1186/s12864-020-06964-6 (PMC7487993; doi:10.1186/s12864-020-06964-6)
Supplement: Supplementary file 2 — Additional file 2: Figure S2. GO distribution of coding sequences found in the Armillaria borealis genome assembly at the molecular function (MF) level based on the GO functional annotation. [file 12864_2020_6964_MOESM2_ESM.pdf]

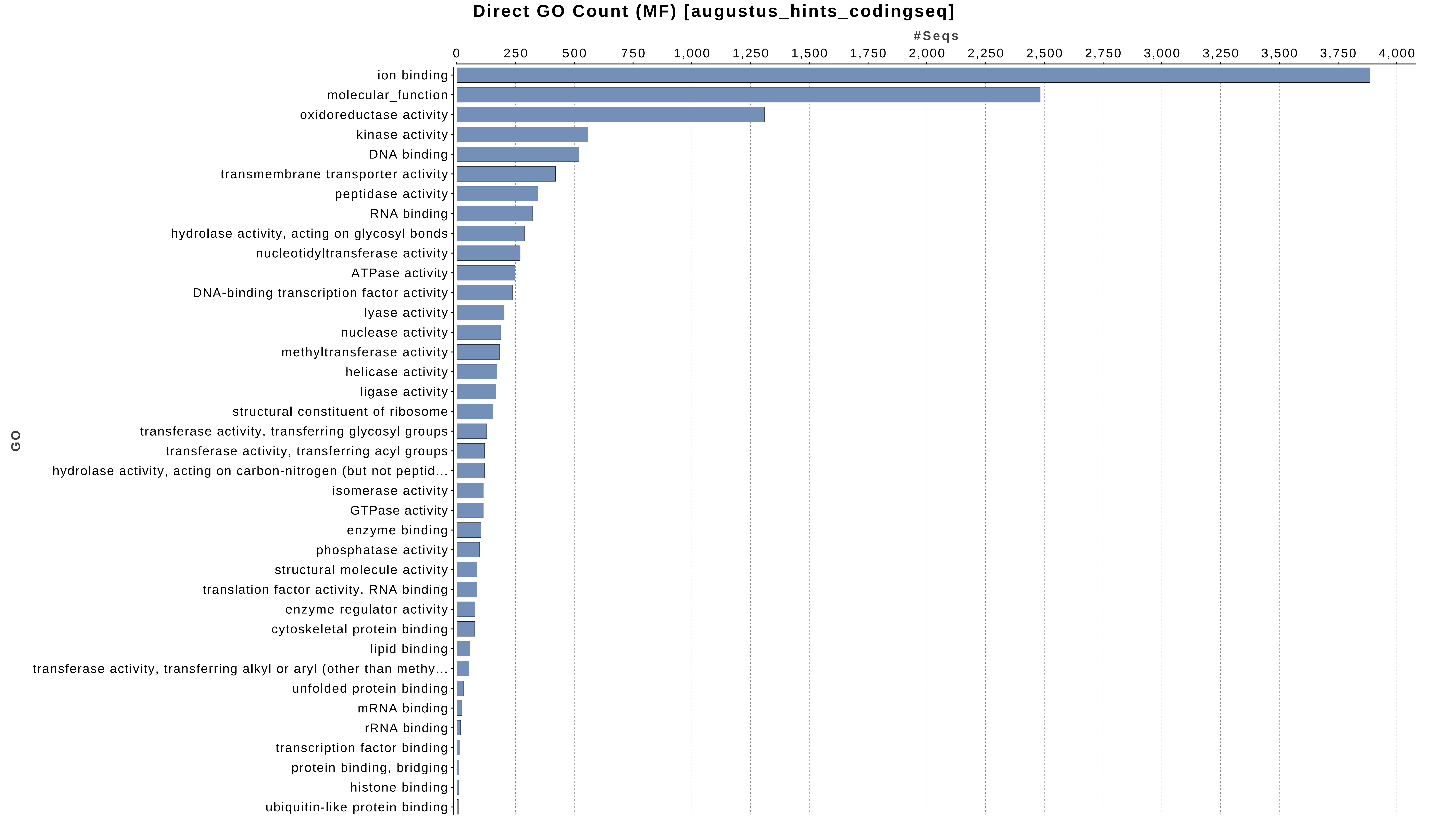
Direct GO Count (MF) [augustus_hints_codingseq]
#Seqs
0
250
500
750
1,000
1,250
1,500
1,750
2,000
2,250
2,500
2,750
3,000
3,250
3,500
3,750
4,000
GO
ion binding
molecular_function
oxidoreductase activity
kinase activity
DNA binding
transmembrane transporter activity
peptidase activity
RNA binding
hydrolase activity, acting on glycosyl bonds
nucleotidyltransferase activity
ATPase activity
DNA-binding transcription factor activity
lyase activity
nuclease activity
methyltransferase activity
helicase activity
ligase activity
structural constituent of ribosome
transferase activity, transferring glycosyl groups
transferase activity, transferring acyl groups
hydrolase activity, acting on carbon-nitrogen (but not peptid...
isomerase activity
GTPase activity
enzyme binding
phosphatase activity
structural molecule activity
translation factor activity, RNA binding
enzyme regulator activity
cytoskeletal protein binding
lipid binding
transferase activity, transferring alkyl or aryl (other than methy...
unfolded protein binding
mRNA binding
rRNA binding
transcription factor binding
protein binding, bridging
histone binding
ubiquitin-like protein binding
